# Factors Associated with Decreased Lean Tissue Index in Patients with Chronic Kidney Disease

**DOI:** 10.3390/nu9050434

**Published:** 2017-04-27

**Authors:** Yi-Wen Wang, Ting-Yun Lin, Ching-Hsiu Peng, Jui-Lin Huang, Szu-Chun Hung

**Affiliations:** 1 Division of Family Medicine, Taipei Tzu Chi Hospital, Buddhist Tzu Chi Medical Foundation, and School of Medicine, Tzu Chi University, Hualien 970, Taiwan; tk7643@hotmail.com; 2 Division of Nephrology, Taipei Tzu Chi Hospital, Buddhist Tzu Chi Medical Foundation, and School of Medicine, Tzu Chi University, Hualien 970, Taiwan; water_h2o_6@hotmail.com (T.-Y.L.); soranos2008@yahoo.com.tw (C.-H.P.); Lancelot_Lien@tw.tdk.com (J.-L.H.)

**Keywords:** bioimpedance, body composition, cardiovascular disease, chronic kidney disease, inflammation, lean tissue index, muscle mass, protein energy wasting

## Abstract

Muscle wasting is common and is associated with increased morbidity and mortality in patients with chronic kidney disease (CKD). However, factors associated with decreased muscle mass in CKD patients are seldom reported. We performed a cross-sectional study of 326 patients (age 65.8 ± 13.3 years) with stage 3–5 CKD who were not yet on dialysis. Muscle mass was determined using the Body Composition Monitor (BCM), a multifrequency bioimpedance spectroscopy device, and was expressed as the lean tissue index (LTI, lean tissue mass/height^2^). An LTI of less than 10% of the normal value (low LTI) indicates muscle wasting. Patients with low LTI (*n* = 40) tended to be diabetic, had significantly higher fat tissue index, urine protein creatinine ratio, and interleukin-6 and tumor necrosis factor-α levels, but had significantly lower serum albumin and hemoglobin levels compared with those with normal LTI. In multivariate linear regression analysis, age, sex, cardiovascular disease, and interleukin-6 were independently associated with LTI. Additionally, diabetes mellitus remained an independent predictor of muscle wasting according to low LTI by multivariate logistic regression analysis. We conclude that LTI has important clinical correlations. Determination of LTI may aid in clinical assessment by helping to identify muscle wasting among patients with stage 3–5 CKD.

## 1. Introduction

Body composition is one of the best indicators of overall health. Muscle wasting is common and progressive in patients with chronic kidney disease (CKD) and has important health consequences [1,2]. The term “sarcopenia”, as defined by the European Working Group on Sarcopenia in Older People (EWGSOP), describes the presence of both low muscle mass and low muscle function (strength or performance) [3]. Recent studies have reported that the prevalence of sarcopenia or muscle wasting among patients with end-stage renal disease (ESRD) on dialysis ranges from 20% to 44% [4,5,6,7], which is significantly higher than in the healthy population.

In adults without CKD, a loss of muscle mass of 1% per year is expected [8,9]. However, in patients with CKD, loss of muscle mass occurs earlier and is much more rapid [10]. Sarcopenia or reduced muscle mass is associated with osteoporosis [11], a higher risk of fracture [12], physical disability [13], functional impairment, hospitalization, increased health costs, and higher mortality in the general population [14,15,16,17]. Recently, it has been shown that muscle wasting is associated with a higher risk of mortality in hemodialysis (HD) patients [5]. Identification and treatment of the underlying cause are required. However, factors associated with decreased muscle mass in non-dialysis CKD patients are seldom reported.

In order to improve the prognosis of CKD patients, sarcopenia should be screened for and detected during mild to moderate stages of CKD, when the complications of reduced muscle mass may be reversible [18]. Bioimpedance spectroscopy is a simple and effective approach for the assessment of body composition. The Body Composition Monitor (BCM, Fresenius Medical Care, Bad Homburg, Germany) is a bedside whole-body bioimpedance spectroscopy device for clinical use. The accuracy of body composition measurements has been validated against available gold standard reference methods including dual energy X-ray absorptiometry (DEXA) [19,20]. Moreover, the safe and accurate use of the BCM has been ensured in CKD patients with and without dialysis [21,22,23]. Therefore, the aim of this study was to determine muscle mass using the BCM device and to identify factors associated with decreased muscle mass in non-dialysis CKD patients.

## 2. Materials and Methods

### 2.1. Design and Participants

This was a cross-sectional study conducted in the Taipei Tzu Chi Hospital, Taiwan. This study complied with the Declaration of Helsinki and was approved by the Institutional Review Board (01-XD13-034). All participants gave written informed consent. Patients with prevalent stage 3–5 CKD who had been seen in outpatient clinics but who were not yet on dialysis were assessed for eligibility for inclusion if they were >18 years of age. CKD was defined on the basis of two (separated by an interval of >3 months) estimated glomerular filtration rate (eGFR) values <60 mL/min per 1.73 m^2^, as calculated by a simplified Modifications of Diet in Renal Disease (MDRD) equation. All patients received multidisciplinary CKD care, focusing on dietary salt and protein restriction, nephrotoxin avoidance, and strict blood pressure and glycemic control. Patients were excluded if they had a cardiac pacemaker or metallic implants, or were amputees. Patients were also excluded if they had malignancies or clinical conditions affecting body composition, such as liver cirrhosis, active inflammatory diseases, or any acute cardiovascular event during the three months before screening for inclusion.

For each patient, a medical history was taken at the time of enrollment. Diabetes mellitus (DM) was defined as the current or past use of insulin and/or oral antidiabetic drugs. Hypertension was defined by a blood pressure ≥140/90 mm Hg or by receiving treatment with anti-hypertensive agents. The definition of cardiovascular disease (CVD) included coronary artery disease, as documented by coronary angiography or a history of myocardial infarction, class III to IV congestive heart failure, or cerebrovascular accidents. Smoking history was defined as any use of tobacco.

### 2.2. Laboratory Measurements

Plasma levels of interleukin-6 (IL-6), tumor necrosis factor α (TNF-α) (R&D Systems, Minneapolis, MN, USA), and N-terminal pro-brain natriuretic peptide (NT-proBNP; Roche Diagnostics, Indianapolis, IN, USA) were determined using commercially available enzyme-linked immunosorbent assay kits according to the manufacturers’ instructions. Serum albumin levels were measured using a bromocresol purple (BCP) assay. Proteinuria was determined based on the urine protein-to-creatinine ratio (UPCR) assessed using the first morning void. Arterial stiffness was obtained by measuring the brachial-ankle pulse wave velocity (baPWV) using a VP-1000 analyzer (Colin Corporation, Komaki, Japan).

### 2.3. Body Composition Measurements

Body composition was assessed using the BCM device. Electrodes were placed on the hand and foot on the nondominant side of the body while the patient was in a supine position, and the results were available within 2 min. The BCM measures body composition by analyzing electrical responses at 50 different frequencies between 5 and 1000 kHz. The use of multiple frequencies enables the calculation of theoretical resistance values at zero and infinite frequencies by fitting a polynomial curve termed the Cole–Cole plot. The validity of the measurement can be assessed by the graphic representation of the curve. Only patients with at least one valid BCM measurement were included in the study.

Input parameters included the patient’s age, sex, height, and weight. Muscle mass was expressed as the lean tissue mass or lean tissue index (LTI, lean tissue mass/height^2^), which was derived from the impedance data based on a three-compartment model (lean tissue mass, adipose tissue mass, and overhydration) [23]. The BCM can distinguish muscle mass from pathologic fluid retention (overhydration). As a result, the sum of lean tissue mass and adipose tissue mass equals the body weight when no excess fluid is present in the body. According to the measured LTI compared with the age- and gender-normalized LTI value, patients with an LTI less than 10% of the normal value were considered to have muscle wasting [5,24]. Patients with an LTI of 10% or greater were considered to have a normal LTI. The BCM device has been validated in a study involving 500 healthy people with the same ethnic background in our study (Taiwanese). Almost all output parameters fit into the same reference ranges set by the Fresenius Medical Care, Bad Homburg, Germany [22,23].

### 2.4. Statistical Analysis

All of the variables were reported as the frequency and percentage for categorical data and as the mean ± SD or median and interquartile range for continuous data with or without a normal distribution, respectively. The study population was further divided into two subgroups according to low (less than 10% of the normal value) or normal LTI. For all comparisons of baseline characteristics between the two subgroups, Student’s *t*-test or the Mann–Whitney *U*-test was used to compare continuous variables. Categorical variables were compared by using a χ^2^ test. Univariate correlations between LTI and potential explanatory variables were assessed by Pearson’s correlation coefficient. Multivariate linear and logistic regression analyses were conducted with the LTI and the presence of muscle wasting (defined as LTI < 10% of the normal value) as dependent variables and potential predictors (Age, sex, DM, CVD, eGFR, and log IL-6) as independent variables. A two-tailed *p*-value < 0.05 was considered statistically significant. Statistical analysis was performed using the computer software Statistical Package for the Social Sciences, version 20 (SPSS, IBM, Armonk, NY, USA).

## 3. Results

### 3.1. Population Characteristics

After the exclusion criteria were applied, 338 clinically stable patients were enrolled in the study. Twelve patients were excluded from the analysis due to loss of follow-up after the initial visit (*n* = 8) or the initiation of chronic dialysis within the first month after enrollment (*n* = 4). The baseline characteristics of the remaining 326 patients, divided according to low or normal LTI, are presented in Table 1. Overall, 45.4% (*n* = 148) of the study population had DM and 23.6% (*n* = 77) had pre-existing CVD. All patients had moderate to severe CKD (mean eGFR 28.8 ± 14.7 mL/min per 1.73 m^2^; *n* = 146 in stage 3; *n* = 107 in stage 4; and *n* = 73 in stage 5). Patients in the low LTI group were more likely to have DM and a smoking history. Furthermore, patients with a low LTI had significantly higher levels of fat tissue index, UPCR, IL-6, and TNF-α but had significantly lower levels of serum albumin and hemoglobin.

### 3.2. Factors Associated with LTI

**Figure 1:**
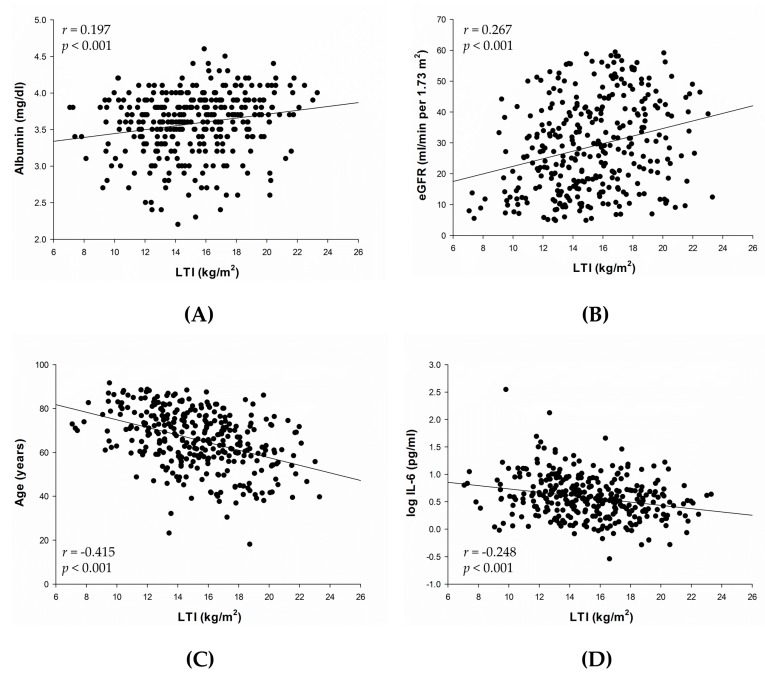
Univariate analysis of the correlations between LTI and serum albumin (**A**); eGFR (**B**); age (**C**); and log IL-6 (**D**). Abbreviations: eGFR, estimated glomerular filtration rate; IL-6, interleukin-6; LTI, lean tissue index.

In univariate analysis, LTI was positively associated with body mass index (*r* = 0.284, *p* < 0.001) and negatively associated with fat tissue index (*r* = −0.432, *p* < 0.001). Correlations between LTI and other variables are presented in Figure 1A–D. LTI was positively correlated with serum albumin (*r* = 0.197, *p* < 0.001) (Figure 1A) and eGFR (*r* = 0.267, *p* < 0.001) (Figure 1B), and negatively correlated with age (*r* = −0.415, *p* < 0.001) (Figure 1C) and log IL-6 (*r* = −0.248, *p* < 0.001) (Figure 1D).

Results of the multivariate linear and logistic regression analyses are presented in Table 2 and Table 3. Stepwise multivariate linear regression analysis showed that age, sex, CVD, and log IL-6 were independently associated with LTI (adjusted *R*^2^ of the model = 0.454) (Table 2). In logistic regression analysis, DM remained an independent predictor of the presence of muscle wasting according to low LTI (Table 3).

## 4. Discussion

We found that LTI was independently associated with age, sex, CVD, and IL-6. In addition, we observed that DM was an independent predictor of muscle wasting, defined as LTI being less than 10% of the normal value in the multivariate logistic regression model. Therefore, LTI has important clinical correlations and may aid in the clinical assessment of patients with stage 3–5 CKD.

Several studies have shown that lean mass decreases with age in older adults without CKD [25,26,27]. Our study showed that LTI was significantly and negatively associated with age, suggesting that age-related muscle wasting also occurs in patients with CKD. Age-related muscle loss may be the result of a disproportionate atrophy of type IIa muscle fibers, decreased synthesis of myosin heavy chain proteins, decline of anabolic hormone levels, loss of innervation, cytokine imbalance, and voluntary inactivity [28]. Undernutrition and decreased physical activity can accelerate age-related muscle loss, and the severity and frequency of sarcopenia increase sharply when patients have several co-morbidities, including osteoporosis, DM, endocrine diseases, neurodegenerative disorders, advanced organ failure, and chronic inflammatory states [3]. Lower LTI values were found in women in our study, consistent with the findings of a previous study that enrolled 14,818 adults using data from the Third National Health and Nutrition Examination Survey (NHANES III) and showed that sarcopenia was greater among older women than among older men [13].

Sarcopenia is common and occurs in all stages of CKD. Foley et al. [29] assessed patients in the NHANES III study and reported a higher prevalence of sarcopenia in patients with lower eGFR. Another cross-sectional study also reported that more advanced stages of CKD are associated with an increased prevalence of sarcopenia [30]. In our study, LTI was positively associated with eGFR, suggesting that preservation of residual renal function may be important to prevent progressive muscle wasting. Protein-energy wasting (PEW) refers to the multiple nutritional and catabolic alterations that occur in CKD [31]. Sarcopenia is a major feature of PEW. The etiology of PEW is multifactorial and involves uremia-induced alterations such as increased energy expenditure, metabolic acidosis, persistent inflammation, and multiple endocrine disorders that result in a state of hypermetabolism leading to excess muscle catabolism [3]. Most of these abnormalities stimulate the ATP-dependent ubiquitin-proteasome system (UPS) pathway, which has been identified as the major pathway in muscle wasting in CKD [32].

We found that DM was an independent predictor for muscle wasting in CKD patients in the present study. Insulin resistance has been identified as one of the most important metabolic challenges in patients with CKD. It is linearly correlated with decline in renal function [33] and is seen in almost all ESRD patients [34]. Furthermore, it is associated with muscle protein degradation, primarily through the UPS pathway. Our results are consistent with previous findings in CKD patients on dialysis. The presence of DM in chronic HD patients is known to increase the risk of protein depletion and the loss of lean body mass [35]. Pupim et al. [36] demonstrated that HD patients with DM had significantly increased muscle protein breakdown compared to non-diabetic HD patients. In a subsequent clinical study [37], it was found that DM is an independent risk factor for muscle wasting in patients with ESRD. Possible mechanisms include concomitant insulin resistance and inflammatory processes. Increased protein loss in the urine and increased energy expenditure in comparison to gender- and age-matched non-diabetic CKD patients further contribute to decreased muscle mass in diabetic CKD patients [38,39].

Low-grade inflammation is common even in earlier stages of CKD, as evidenced by increased circulating levels of inflammatory markers such as C-reactive protein (CRP), IL-6, and TNF-α. After multivariate adjustment, IL-6 remained an independent predictor of LTI in our study, suggesting that the development of decreased muscle mass in CKD may be mediated by chronic inflammation. Muscle mass in long-term HD patients is inversely associated with inflammatory markers such as serum IL-6 and CRP levels [40,41,42,43]. Several mechanisms for inflammation-induced muscle wasting have been described. For example, TNF-α enhances muscle wasting through the inhibition of myogenic differentiation by activating the nuclear factor-kappa B (NFκB) pathway [44]. Moreover, inflammation contributes to CVD as well as PEW. The concurrence of high IL-6 levels and CVD in patients with decreased LTI in our study corroborates the findings of Stenvinkel et al., who showed a strong association between malnutrition, inflammation, and atherosclerosis (MIA syndrome) in patients with advanced CKD [45].

Some limitations of our study should be acknowledged. Because of the cross-sectional design of the study, causality cannot be established, and there remain some potential unknown or unmeasured confounding factors associated with LTI. Dietary intake was not assessed in this study. Decreased protein intake may be a risk factor for loss of lean mass in patients with CKD and should be studied further. The strengths of this study are that the BCM device is portable and relatively inexpensive, and that measurements are non-invasive, simple, rapid, and highly reproducible [46]. Moreover, the BCM can distinguish muscle mass from pathologic fluid retention in CKD, and provide a more accurate estimate of body composition. Another strength is that we were able to identify associating factors for LTI that have not previously been reported in non-dialysis CKD patients.

## 5. Conclusions

A better understanding of the factors associated with decreased muscle mass is needed to develop preventive strategies for muscle wasting in patients with CKD. Muscle mass, as measured by the BCM and expressed as the LTI, is strongly associated with age, sex, DM, CVD, and IL-6. The BCM may help to identify a high-risk group with decreased muscle mass among patients with stage 3–5 CKD. Control of inflammation and underlying comorbidities such as DM and CVD, in addition to appropriate nutritional, pharmalogical, and exercise interventions [47,48], may have protective effects on muscle mass and benefit CKD patients with muscle wasting. Nevertheless, further studies are warranted to confirm our findings.

## Figures and Tables

**Table 1:** Characteristics of CKD patients categorized according to low or normal LTI. Abbreviations: baPWV, brachial-ankle pulse wave velocity; BP, blood pressure; CKD, chronic kidney disease; CVD, cardiovascular disease; DM, diabetes mellitus; eGFR, estimated glomerular filtration rate; hs-CRP, high-sensitivity C-reactive protein; IL-6, interleukin-6; LTI, lean tissue index; RAS, renin-angiotensin system; TNF-α, tumor necrosis factor α; UPCR, urine protein creatinine ratio.

Characteristic	LTI (kg/m^2^)		*p* Value
	<10% (*n* = 40)	≥10% (*n* = 286)	
Age (years)	63.9 ± 11.8	66.1 ± 13.5	0.331
Male sex, *n* (%)	26 (63.4%)	198 (69.5%)	0.434
Smoking history, *n* (%)	14 (34.1%)	53 (18.6%)	0.021
DM, *n* (%)	26 (63.4%)	122 (42.8%)	0.013
CVD, *n* (%)	11 (26.8%)	66 (23.2%)	0.605
Systolic BP (mmHg)	139.2 ± 16.4	137.4 ± 17.3	0.530
baPWV (m/s)	16.5 ± 2.8	15.9 ± 3.1	0.262
RAS blockers, *n* (%)	20 (48.8%)	176 (61.8%)	0.113
Statin, *n* (%)	15 (36.6%)	71 (24.9%)	0.113
Body mass index (kg/m^2^)	24.8 ± 4.9	26.0 ± 4.0	0.081
Fat tissue index (kg/m^2^)	12.1 ± 5.1	9.4 ± 4.1	0.000
eGFR (mL/min/1.73 m^2^)	25.3 ± 14.6	29.4 ± 14.7	0.098
UPCR (g/g)	1.40 (0.55–4.21)	0.81 (0.30–2.22)	0.020
Albumin (g/dL)	3.4 ± 0.4	3.6 ± 0.4	0.007
Fasting glucose (mg/dL)	122 ± 40	121 ± 42	0.816
Total cholesterol (mg/dL)	173 ± 45	175 ± 40	0.791
Triglyceride (mg/dL)	163 ± 80	164 ± 119	0.958
hs-CRP (mg/L)	3.8 (1.5–10.8)	3.9 (1.3–9.6)	0.910
IL-6 (pg/mL)	5.86 (2.82–8.86)	3.42 (2.04–5.41)	0.017
TNF-α (pg/mL)	8.25 (5.79–11.23)	6.45 (4.47–9.15)	0.002
Hemoglobin (g/dL)	11.0 ± 1.9	12.0 ± 2.1	0.011

**Table 2:** Stepwise multivariate linear regression model identifying determinants of LTI. Abbreviations: CVD, cardiovascular disease; IL-6, interleukin-6; LTI, lean tissue index.

Variable	Standard Error	Beta Coefficient	*t*	*p* Value
Age	0.010	−0.358	−8.360	0.000
Male sex	0.285	0.505	12.225	0.000
CVD	0.318	−0.085	−2.008	0.045
log IL-6 (pg/mL)	0.355	−0.104	−2.401	0.017

**Table 3:** Multivariate logistic regression model for patients with low LTI (<10% of the normal value). Abbreviations: CI, confidence interval; CVD, cardiovascular disease; DM, diabetes mellitus; eGFR, estimated glomerular filtration rate; IL-6, interleukin-6; LTI, lean tissue index; OR, odds ratio.

Variable	OR	95% CI	*p* Value
Age	0.979	0.954 to 1.006	0.125
Male sex	0.969	0.469 to 2.005	0.933
DM	2.058	1.002 to 4.226	0.049
CVD	0.909	0.410 to 2.017	0.815
eGFR (mL/min/1.73 m^2^)	0.981	0.957 to 1.006	0.136
log IL-6 (pg/mL)	2.032	0.858 to 4.814	0.107
